# 2-(1-Methyl­ethoxy)-5-nitro­phenyl *N*-methyl­carbamate

**DOI:** 10.1107/S1600536808040622

**Published:** 2008-12-10

**Authors:** Guang-Ming Sang, Shi-Neng Luo, Jian-Guo Lin, Hai-Lin Yang, Yong-Mei Xia

**Affiliations:** a School of Chemical and Material Engineering, Jiangnan University, Wuxi 214122, People’s Republic of China; b The Key Laboratory of Nuclear Medicine, Ministry of Health, Jiangsu Institute of Nuclear Medicine, Wuxi 214063, People’s Republic of China; c The Key Laboratory of Industrial Biotechnology, Jiangnan University, Wuxi 214122, People’s Republic of China

## Abstract

In the title compound, C_11_H_14_N_2_O_5_, the nitro group is approximately coplanar with the benzene ring, making a dihedral angle of 4.26 (17)°. The dihedral angle between the methyl­carbamate group and the benzene ring is 72.47 (6)°. There is a strong inter­molecular N—H⋯O hydrogen bond between the N and O atoms from adjacent methyl­carbamate groups, forming a one-dimensional network along the *a* axis.

## Related literature

For general background, see: Wang *et al.* (1998 ▶); Moreno *et al.* (2001 ▶). For related structures, see: Czugler & Kalman (1975 ▶); Xu *et al.* (2005 ▶). For the synthesis, see: Allan *et al.* (1926 ▶).
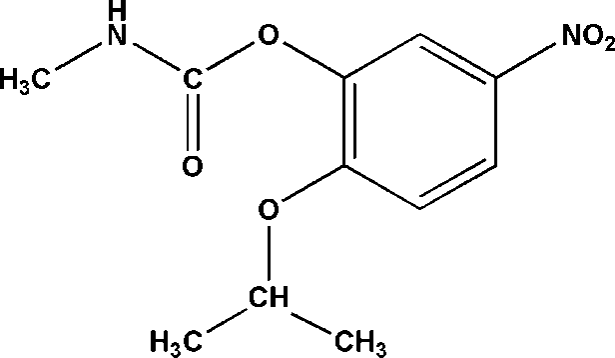


## Experimental

### 

#### Crystal data

C_11_H_14_N_2_O_5_
*M* *_r_* = 254.24
Triclinic, 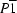

*a* = 5.034 (2) Å
*b* = 10.4221 (16) Å
*c* = 12.6319 (12) Å
α = 91.361 (3)°
β = 97.492 (2)°
γ = 94.6930 (10)°
*V* = 654.5 (3) Å^3^
*Z* = 2
Mo *K*α radiation
μ = 0.10 mm^−1^
*T* = 291 (2) K
0.30 × 0.26 × 0.24 mm

#### Data collection

Bruker SMART APEX CCD diffractometer
Absorption correction: multi-scan (*SADABS*; Bruker, 2001 ▶) *T*_min_ = 0.97, *T*_max_ = 0.98
7186 measured reflections
3172 independent reflections
2005 reflections with *I* > 2σ(*I*)
*R*_int_ = 0.038

#### Refinement

*R*[*F* ^2^ > 2σ(*F* ^2^)] = 0.049
*wR*(*F* ^2^) = 0.105
*S* = 1.03
3172 reflections
167 parameters
H-atom parameters constrained
Δρ_max_ = 0.25 e Å^−3^
Δρ_min_ = −0.21 e Å^−3^

### 

Data collection: *SMART* (Bruker, 2001 ▶); cell refinement: *SAINT* (Bruker, 2001 ▶); data reduction: *SAINT*; program(s) used to solve structure: *SHELXTL* (Sheldrick, 2008 ▶); program(s) used to refine structure: *SHELXTL*; molecular graphics: *SHELXTL*; software used to prepare material for publication: *SHELXTL*.

## Supplementary Material

Crystal structure: contains datablocks global, I. DOI: 10.1107/S1600536808040622/fj2175sup1.cif

Structure factors: contains datablocks I. DOI: 10.1107/S1600536808040622/fj2175Isup2.hkl

Additional supplementary materials:  crystallographic information; 3D view; checkCIF report

## Figures and Tables

**Table 1:** Hydrogen-bond geometry (Å, °)

*D*—H⋯*A*	*D*—H	H⋯*A*	*D*⋯*A*	*D*—H⋯*A*
N2—H2*A*⋯O5^i^	0.86	2.05	2.788 (2)	143

Symmetry code: (i) 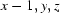.

**Fig. 1.**
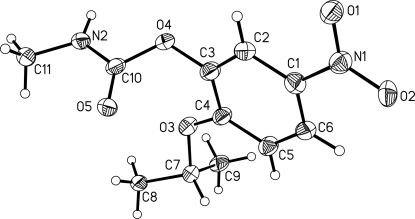
The molecular structure of (I), with atom labels and 30% probability displacement ellipsoids for non-H atoms.

**Fig. 2.**
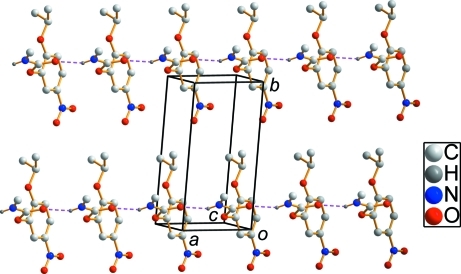
Perspective view of the supramolecular network along a axis built from strong intermolecular N—H···O hydrogen bonds (dashed lines). H atoms not involved in hydrogen bonds have been omitted.

## supplementary crystallographic information

### Comment

2-(1-Methylethoxy)phenyl methylcarbamate (Trade name: Propoxur) is an important economical insecticide. It is widely used to control agricultural and household insect pests due to its low toxicity to mammals and other vertebrates (Wang *et al.*, 1998; Moreno *et al.*, 2001). Immunoassay is one of effective analytical methods of determining the residua of the methylcarbamate pesticide propoxur. Propoxur, like most pesticides, is a small and simple organic molecule, which lacks a functional group (amido or carboxylic acid) for coupling to proteins and is non immunogenic by itself. Therefore, it is necessary to synthesis hapten resembling as much as possible the structural and electronic distribution of propoxur for the production of highaffinity antibodies (Moreno *et al.*, 2001). With this idea in mind, we intend to synthesis 5-amino-2-(1-methylethoxy)phenyl methylcarbamate. As a vital intermediate compound for the stepwise reactions of hapten synthesis, the synthesis and crystal structure of the title compound has been reported herein.

In the title compound (I) (Fig. 1), C_11_H_14_N_2_O_5_, the nitro group is approximately coplanar with the phenyl ring [dihedral angle = 4.26 (17)°]. All the nonhydrogen atoms in the methylcarbamate group are almost in a plane, and the dihedral angle between methylcarbamate group and phenyl is 72.47 (6)°. There is a strong N—H···O intermolecular hydrogen bond between the N2 atom and O5 atom from adjacent methylcarbamate groups (Table 1). And the crystal structure is stabilized by these strong hydrogen bond interactions to form one-dimensional supramolecular network along *a* axis (Table 1 and Fig. 2).

### Experimental

The title compound (I) was synthesized as follows (Allan *et al.*, 1926): Nitric acid (25 ml, *d* 1.42, 0.6 mol) was added to a solution of 2-(1-methylethoxy)-phenyl methylcarbamate (20.9 g, 0.1 mol) in acetic acid (30 ml), and the mixture was heated on the oil-bath until the onset of a vigorous reaction was manifested by the copious evolution of red fumes and temperature rising to around 100 °C. Then, the reaction mixture was heated on this condition for 3 h, poured into cool water, and stirred for 30 min. After filtering, washing with water and drying in vacuum, a white powder was then obtained (yield: 75%). mp 120–121 °C. The title compound was recrystallized from ethanol solvent; colourless block-shaped crystals were formed after several days (yield 58%). Analysis calculated for C_11_H_14_N_2_O_5_: C 51.97, H 5.55, N 11.02%; found: C 51.92, H 5.49, N 11.08%.

### Refinement

H atoms bonded to N atom was located in a difference map and refined with distance restraints of N—H = 0.86 Å, and with *U*_iso_(H) = 1.2*U*_eq_(N). Other H atoms were positioned geometrically and refined using a riding model (including free rotation about the ethanol C—C bond), with C—H = 0.93–0.98 Å and with *U*_iso_(H) = 1.2 (1.5 for methyl groups) times *U*_eq_(C).

### Crystal data

**Table:**

C_11_H_14_N_2_O_5_	*Z* = 2
*M**_r_* = 254.24	*F*(000) = 268
Triclinic, *P*1	*D*_x_ = 1.290 Mg m^−^^3^
Hall symbol: -P 1	Mo *K*α radiation, λ = 0.71073 Å
*a* = 5.034 (2) Å	Cell parameters from 825 reflections
*b* = 10.4221 (16) Å	θ = 2.1–25.4°
*c* = 12.6319 (12) Å	µ = 0.10 mm^−^^1^
α = 91.361 (3)°	*T* = 291 K
β = 97.492 (2)°	Block, colourless
γ = 94.693 (1)°	0.30 × 0.26 × 0.24 mm
*V* = 654.5 (3) Å^3^	

### Data collection

**Table:**

Bruker SMART APEX CCD diffractometer	3172 independent reflections
Radiation source: sealed tube	2005 reflections with *I* > 2σ(*I*)
graphite	*R*_int_ = 0.038
φ and ω scans	θ_max_ = 28.0°, θ_min_ = 1.6°
Absorption correction: multi-scan (*SADABS*; Bruker, 2001)	*h* = −6→6
*T*_min_ = 0.97, *T*_max_ = 0.98	*k* = −13→13
7186 measured reflections	*l* = −10→16

### Refinement

**Table:**

Refinement on *F*^2^	Secondary atom site location: difference Fourier map
Least-squares matrix: full	Hydrogen site location: inferred from neighbouring sites
*R*[*F*^2^ > 2σ(*F*^2^)] = 0.049	H-atom parameters constrained
*wR*(*F*^2^) = 0.105	*w* = 1/[σ^2^(*F*_o_^2^) + (0.04*P*)^2^] where *P* = (*F*_o_^2^ + 2*F*_c_^2^)/3
*S* = 1.03	(Δ/σ)_max_ < 0.001
3172 reflections	Δρ_max_ = 0.25 e Å^−^^3^
167 parameters	Δρ_min_ = −0.21 e Å^−^^3^
0 restraints	Extinction correction: *SHELXTL* (Sheldrick, 2008), Fc^*^=kFc[1+0.001xFc^2^λ^3^/sin(2θ)]^-1/4^
Primary atom site location: structure-invariant direct methods	Extinction coefficient: 0.015 (3)

### Special details

**Table:**

Geometry. All e.s.d.'s (except the e.s.d. in the dihedral angle between two l.s. planes) are estimated using the full covariance matrix. The cell e.s.d.'s are taken into account individually in the estimation of e.s.d.'s in distances, angles and torsion angles; correlations between e.s.d.'s in cell parameters are only used when they are defined by crystal symmetry. An approximate (isotropic) treatment of cell e.s.d.'s is used for estimating e.s.d.'s involving l.s. planes.
Refinement. Refinement of *F*^2^ against ALL reflections. The weighted *R*-factor *wR* and goodness of fit *S* are based on *F*^2^, conventional *R*-factors *R* are based on *F*, with *F* set to zero for negative *F*^2^. The threshold expression of *F*^2^ > σ(*F*^2^) is used only for calculating *R*-factors(gt) *etc*. and is not relevant to the choice of reflections for refinement. *R*-factors based on *F*^2^ are statistically about twice as large as those based on *F*, and *R*- factors based on ALL data will be even larger.

### Fractional atomic coordinates and isotropic or equivalent isotropic displacement parameters (Å^2^)

**Table:**

	*x*	*y*	*z*	*U*_iso_*/*U*_eq_	
C1	0.6429 (3)	1.05070 (14)	0.65269 (13)	0.0418 (3)	
C2	0.4552 (3)	1.04573 (14)	0.72651 (12)	0.0408 (3)	
H2	0.4010	1.1205	0.7557	0.049*	
C3	0.3576 (3)	0.92824 (15)	0.75300 (12)	0.0401 (3)	
C4	0.4281 (3)	0.81480 (14)	0.70606 (12)	0.0404 (3)	
C5	0.6183 (3)	0.82349 (14)	0.63360 (12)	0.0410 (3)	
H5	0.6732	0.7492	0.6039	0.049*	
C6	0.7215 (3)	0.94193 (14)	0.60731 (12)	0.0412 (3)	
H6	0.8450	0.9487	0.5586	0.049*	
C7	0.4126 (3)	0.58077 (15)	0.71348 (12)	0.0419 (3)	
H7	0.6049	0.5905	0.7068	0.050*	
C8	0.3506 (4)	0.49615 (16)	0.80518 (15)	0.0503 (4)	
H8A	0.4181	0.5406	0.8719	0.075*	
H8B	0.4351	0.4173	0.8004	0.075*	
H8C	0.1596	0.4772	0.8011	0.075*	
C9	0.2464 (4)	0.52696 (15)	0.61402 (13)	0.0475 (4)	
H9A	0.0596	0.5236	0.6231	0.071*	
H9B	0.2939	0.4417	0.5990	0.071*	
H9C	0.2783	0.5809	0.5556	0.071*	
C10	0.2341 (3)	0.86019 (14)	0.91676 (12)	0.0379 (3)	
C11	0.0362 (3)	0.77353 (16)	1.06731 (13)	0.0446 (4)	
H11A	0.1398	0.7002	1.0669	0.067*	
H11B	−0.1414	0.7466	1.0827	0.067*	
H11C	0.1217	0.8356	1.1211	0.067*	
N1	0.7563 (3)	1.17674 (12)	0.62443 (11)	0.0435 (3)	
N2	0.0186 (3)	0.83114 (12)	0.96365 (10)	0.0403 (3)	
H2A	−0.1364	0.8468	0.9318	0.048*	
O1	0.6715 (2)	1.27246 (10)	0.66209 (9)	0.0473 (3)	
O2	0.9315 (2)	1.18325 (10)	0.56528 (9)	0.0446 (3)	
O3	0.3060 (2)	0.70444 (10)	0.73769 (9)	0.0420 (3)	
O4	0.1650 (2)	0.91925 (10)	0.82168 (9)	0.0418 (3)	
O5	0.4638 (2)	0.84188 (10)	0.95050 (9)	0.0421 (3)	

### Atomic displacement parameters (Å^2^)

**Table:**

	*U*^11^	*U*^22^	*U*^33^	*U*^12^	*U*^13^	*U*^23^
C1	0.0464 (9)	0.0377 (7)	0.0423 (8)	0.0040 (6)	0.0085 (7)	0.0037 (6)
C2	0.0388 (8)	0.0429 (8)	0.0423 (8)	0.0116 (6)	0.0067 (6)	0.0029 (6)
C3	0.0387 (8)	0.0459 (8)	0.0372 (8)	0.0083 (6)	0.0084 (6)	−0.0029 (6)
C4	0.0410 (8)	0.0418 (8)	0.0404 (8)	0.0090 (6)	0.0110 (6)	−0.0028 (6)
C5	0.0468 (9)	0.0389 (7)	0.0389 (8)	0.0066 (6)	0.0111 (6)	−0.0056 (6)
C6	0.0428 (8)	0.0432 (8)	0.0396 (8)	0.0111 (6)	0.0081 (6)	0.0020 (6)
C7	0.0422 (8)	0.0495 (8)	0.0368 (8)	0.0112 (7)	0.0107 (6)	0.0048 (6)
C8	0.0526 (10)	0.0487 (9)	0.0528 (10)	0.0121 (7)	0.0124 (8)	0.0120 (7)
C9	0.0516 (10)	0.0448 (9)	0.0474 (9)	0.0104 (7)	0.0098 (7)	−0.0148 (7)
C10	0.0319 (7)	0.0431 (8)	0.0404 (8)	0.0099 (6)	0.0081 (6)	−0.0047 (6)
C11	0.0439 (9)	0.0507 (9)	0.0422 (9)	0.0122 (7)	0.0101 (7)	0.0093 (7)
N1	0.0403 (7)	0.0433 (7)	0.0477 (8)	0.0027 (5)	0.0091 (6)	0.0016 (5)
N2	0.0333 (6)	0.0458 (7)	0.0453 (8)	0.0124 (5)	0.0118 (5)	0.0096 (5)
O1	0.0508 (7)	0.0413 (6)	0.0529 (7)	0.0042 (5)	0.0191 (5)	0.0017 (5)
O2	0.0530 (7)	0.0433 (6)	0.0387 (6)	−0.0037 (5)	0.0141 (5)	0.0074 (4)
O3	0.0415 (6)	0.0427 (6)	0.0437 (6)	0.0036 (4)	0.0136 (5)	−0.0016 (4)
O4	0.0447 (6)	0.0411 (5)	0.0450 (6)	0.0169 (5)	0.0170 (5)	0.0039 (4)
O5	0.0360 (6)	0.0487 (6)	0.0449 (6)	0.0140 (5)	0.0097 (5)	0.0127 (5)

### Geometric parameters (Å, °)

**Table:**

C1—C6	1.368 (2)	C8—H8A	0.9600
C1—C2	1.410 (2)	C8—H8B	0.9600
C1—N1	1.4600 (19)	C8—H8C	0.9600
C2—C3	1.348 (2)	C9—H9A	0.9600
C2—H2	0.9300	C9—H9B	0.9600
C3—O4	1.3818 (18)	C9—H9C	0.9600
C3—C4	1.402 (2)	C10—O5	1.2107 (18)
C4—O3	1.3530 (19)	C10—N2	1.3195 (18)
C4—C5	1.408 (2)	C10—O4	1.3797 (19)
C5—C6	1.365 (2)	C11—N2	1.4487 (19)
C5—H5	0.9300	C11—H11A	0.9600
C6—H6	0.9300	C11—H11B	0.9600
C7—O3	1.4766 (18)	C11—H11C	0.9600
C7—C9	1.486 (2)	N1—O1	1.2263 (17)
C7—C8	1.521 (2)	N1—O2	1.2270 (17)
C7—H7	0.9800	N2—H2A	0.8600
			
C6—C1—C2	122.26 (14)	C7—C8—H8C	109.5
C6—C1—N1	119.34 (15)	H8A—C8—H8C	109.5
C2—C1—N1	118.40 (14)	H8B—C8—H8C	109.5
C3—C2—C1	117.29 (14)	C7—C9—H9A	109.5
C3—C2—H2	121.4	C7—C9—H9B	109.5
C1—C2—H2	121.4	H9A—C9—H9B	109.5
C2—C3—O4	119.06 (13)	C7—C9—H9C	109.5
C2—C3—C4	122.00 (15)	H9A—C9—H9C	109.5
O4—C3—C4	118.72 (13)	H9B—C9—H9C	109.5
O3—C4—C3	115.20 (14)	O5—C10—N2	126.72 (15)
O3—C4—C5	125.76 (13)	O5—C10—O4	122.87 (14)
C3—C4—C5	119.03 (14)	N2—C10—O4	110.40 (13)
C6—C5—C4	119.41 (14)	N2—C11—H11A	109.5
C6—C5—H5	120.3	N2—C11—H11B	109.5
C4—C5—H5	120.3	H11A—C11—H11B	109.5
C5—C6—C1	119.92 (15)	N2—C11—H11C	109.5
C5—C6—H6	120.0	H11A—C11—H11C	109.5
C1—C6—H6	120.0	H11B—C11—H11C	109.5
O3—C7—C9	106.04 (13)	O1—N1—O2	122.72 (13)
O3—C7—C8	104.34 (12)	O1—N1—C1	117.78 (13)
C9—C7—C8	108.30 (15)	O2—N1—C1	119.50 (13)
O3—C7—H7	112.5	C10—N2—C11	121.78 (13)
C9—C7—H7	112.5	C10—N2—H2A	119.1
C8—C7—H7	112.5	C11—N2—H2A	119.1
C7—C8—H8A	109.5	C4—O3—C7	118.97 (12)
C7—C8—H8B	109.5	C10—O4—C3	116.28 (12)
H8A—C8—H8B	109.5		
			
C6—C1—C2—C3	1.2 (2)	C2—C1—N1—O1	−3.8 (2)
N1—C1—C2—C3	−178.66 (15)	C6—C1—N1—O2	−4.2 (2)
C1—C2—C3—O4	−177.36 (14)	C2—C1—N1—O2	175.73 (15)
C1—C2—C3—C4	−2.9 (2)	O5—C10—N2—C11	1.4 (2)
C2—C3—C4—O3	−177.54 (14)	O4—C10—N2—C11	−177.13 (13)
O4—C3—C4—O3	−3.1 (2)	C3—C4—O3—C7	−165.57 (13)
C2—C3—C4—C5	3.7 (3)	C5—C4—O3—C7	13.1 (2)
O4—C3—C4—C5	178.15 (14)	C9—C7—O3—C4	−96.64 (16)
O3—C4—C5—C6	178.71 (15)	C8—C7—O3—C4	149.11 (14)
C3—C4—C5—C6	−2.7 (2)	O5—C10—O4—C3	15.7 (2)
C4—C5—C6—C1	1.1 (3)	N2—C10—O4—C3	−165.71 (12)
C2—C1—C6—C5	−0.3 (3)	C2—C3—O4—C10	−118.54 (16)
N1—C1—C6—C5	179.56 (14)	C4—C3—O4—C10	66.83 (18)
C6—C1—N1—O1	176.26 (14)		

### Hydrogen-bond geometry (Å, °)

**Table:**

*D*—H···*A*	*D*—H	H···*A*	*D*···*A*	*D*—H···*A*
N2—H2A···O5^i^	0.86	2.05	2.788 (2)	143

Symmetry codes: (i) *x*−1, *y*, *z*.
